# Dual energy X-ray absorptiometry-measured fat mass and lean mass indices and cardiometabolic diseases in elderly Japanese men: the Fujiwara-kyo Osteoporosis Risk in Men (FORMEN) study

**DOI:** 10.1265/ehpm.25-00133

**Published:** 2025-07-25

**Authors:** Katsuyasu Kouda, Yuki Fujita, Yuki Murakami, Kumiko Ohara, Takahiro Tachiki, Junko Tamaki, Jong-Seong Moon, Etsuko Kajita, Akemi Nitta, Nami Imai, Kazuhiro Uenishi, Masayuki Iki

**Affiliations:** 1 Department of Hygiene and Public Health, Faculty of Medicine, Kansai Medical University, 2-5-1 Shin-machi, Hirakata, Osaka 573-1010, Japan; 2 Department of Epidemiology for Community Health and Medicine, Kyoto Prefectural University of Medicine, 465 Kajii-cho, Kawaramachi-Hirokoji, Kamigyo, Kyoto, Kyoto 602-8566, Japan; 3 Department of Nursing, Faculty of Nursing, University of Kochi, 2751-1 Ike, Kochi, Kochi 781-8515, Japan; 4 Department of Hygiene and Public Health, Faculty of Medicine, Osaka Medical and Pharmaceutical University, 2-7 Daigakumachi, Takatsuki, Osaka 569-8686, Japan; 5 Department of Nursing, Kio University, 4-2-2 Umami-naka, Koryo-cho, Nara 635-0832, Japan; 6 Nagoya University, Furo-cho, Chikusa-ku, Nagoya 464-8601, Japan; 7 Department of Pharmaceutical and Medical Business Sciences, Nihon Pharmaceutical University, 10281 Komuro, Ina-machi, Kitaadachi-gun, Saitama 362-0806, Japan; 8 Laboratory of Physiological Nutrition, Kagawa Nutrition University, 3-9-21 Chiyoda, Sakado, Saitama 350-0288, Japan

**Keywords:** Body composition, Cardiometabolic risk factor, Densitometry, Epidemiology

## Abstract

**Background:**

High visceral fat mass (FM) is associated with a high risk of cardiometabolic morbidity. Meanwhile, loss of skeletal muscle (lean mass, LM) has been suggested to contribute to metabolic diseases.

**Methods:**

We investigated associations between cardiometabolic diseases and dual energy X-ray absorptiometry (DXA)-measured body composition indices, including the FM index (FM/height^2^), percent body fat, trunk-to-appendicular fat ratio (TAR), trunk-to-leg fat ratio (TLR), LM index (LM/height^2^) and FM-to-LM ratio in 595 community-dwelling elderly Japanese men (mean age, 74 years; standard deviation, 6; range, 65 to 94). Hypertension was identified as high blood pressure and/or the use of antihypertensive drugs. Diabetes was identified as high hemoglobin A1c and/or the use of antidiabetic drugs. The ability of DXA-based indices to discriminate between the presence and absence of cardiometabolic diseases was evaluated using area under the curve (AUC) calculated by receiver operating characteristic curve analysis.

**Results:**

Body mass index, FM index, percent body fat, TAR, TLR and FM-to-LM ratio were significantly associated with hypertension (P < 0.05). TAR and TLR, but not body mass index, FM index, percent body fat, LM index and FM-to-LM ratio, showed significant positive associations with diabetes. The AUC for the LM index was significantly lower than those for the FM index, percent body fat and FM-to-LM ratio. No associations were observed between the LM index and hypertension, dyslipidemia and diabetes.

**Conclusion:**

The association between cardiometabolic function and LM, which includes skeletal muscle, may not be as pronounced or stronger than associations between cardiometabolic function and FM. Further detailed studies are needed to clarify how skeletal muscle contributes to cardiometabolic disease.

## Background

Obesity is generally defined as an excess of body fat [1]. The most commonly used index for obesity is body mass index (BMI), but there is substantial variation in regional fat accumulation at the same BMI [1]. Metabolic consequences of obesity vary by the distribution of adipose tissue [2]. Findings from epidemiological studies have shown that visceral adipose tissue is an independent risk marker of cardiovascular and metabolic morbidity and mortality, and there is evidence suggesting that ectopic fat deposition, including hepatic and epicardial fat, might contribute to increased cardiometabolic risk [3]. Japanese men, who are less obese but have larger areas of visceral adipose tissue than Caucasian men, are more susceptible to metabolic disorders [4, 5]. In contrast, high lower body FM (gluteofemoral obesity) is suggested to be associated with a low risk of cardiovascular disease and type 2 diabetes mellitus when adjusted for overall fat mass [6]. The protective effects of lower body subcutaneous adiposity are linked to its depot function as a “metabolic sink,” receiving and sequestering excess lipids [7]. The loss of adipogenic and storage capacity in the subcutaneous depot accelerates lipid deposition in the visceral depot, and expansion of visceral adipose tissue, which accelerates low-grade inflammation, is a key driver of insulin resistance [8]. Thus, excess subcutaneous fat may represent a helpful energy reserve for the prevention of cardiometabolic diseases [1]. An experimental study reported that fatless mice are severely insulin-resistant, and that upon transplantation of fat tissue into these mice, triglyceride contents in muscle and liver tissues returned to normal as did insulin signaling and action [9]. The effect of surgical implantation of adipose tissue to reverse diabetes in lipoatrophic mice has also been reported [10].

Skeletal muscle has many metabolic functions (i.e., glucose, insulin and protein metabolism, inflammation, oxidative stress, hormone status) and is an emerging factor for cardiovascular diseases [11]. As the regulator of glucose homeostasis, skeletal muscle is responsible for 80% of postprandial glucose uptake from the circulation [12]. Moreover, skeletal muscle influences protein metabolism throughout the body, serving as a reservoir of amino acids [13]. Muscle-derived secretory proteins, referred to as myokines, mediate interactions between skeletal muscle mass and other organs such as the liver, adipose tissue, pancreas, bone and the cardiovascular system [14]. Several studies have suggested that the loss of skeletal muscle mass may contribute to the development of metabolic diseases [14].

Dual energy X-ray absorptiometry (DXA) is considered one of the most versatile imaging techniques used to evaluate fat mass (FM) and lean mass (LM) [15]. The precision of DXA measurements is excellent [16], with negligible radiation exposure (4–5 µSv, which is even lower than the natural background of 2.4 mSv/year) [17]. DXA can provide both whole-body and regional FM, as well as whole-body and regional LM [15]. Since trunk FM includes visceral fat, and LM includes skeletal muscle, body composition indices such as the FM index (FM/height^2^), percent body fat, trunk-to-appendicular fat ratio (TAR), trunk-to-leg fat ratio (TLR), LM index (LM/height^2^) and FM-to-LM ratio can be used to evaluate cardiometabolic risk status. These DXA-based indices are classified as “weight-based measures” (e.g., FM index) and “fat distribution-based measures” (e.g., TAR and TLR) [18].

In a previous Fujiwara-kyo Osteoporosis Risk in Men (FORMEN)-related study, we demonstrated that TAR and TLR are associated with cardiometabolic risk factors independently of whole-body FM in elderly Japanese men who have a high prevalence of hypertension and diabetes [19]. However, there is little information regarding the detailed characteristics of each DXA-based index, especially for LM. Therefore, in the present study, we examined associations between DXA-measured body composition indices including LM and cardiometabolic diseases in community-dwelling elderly Japanese men of the FORMEN study.

## Methods

### Study population

The FORMEN study is a community-based single-center prospective cohort study in Nara Prefecture, Japan, consisting of the first cohort started in 2007 [20] and the second cohort started in 2019 [19]. Participants included men aged ≥65 years who lived at home, walked without assistance from another person, and provided self-reported information and written informed consent. The source population of the present study consists of 599 men who participated in the baseline survey of the second cohort. After excluding one man with missing blood test values, one man with missing DXA-measured body composition values, and two men with incomplete data for DXA-measured body composition, we analyzed 595 men (mean age, 74 years; standard deviation, 6; range, 65 to 94) in the present cross-sectional study.

### Body composition and body size measurements

Whole-body and regional body composition including FM and LM were measured with a single DXA scanner (QDR-4500A; Hologic Inc., Bedford, MA, USA) equipped in a single examination car. The same experienced radiologic technician performed quality controls of the DXA scanner and measurements for all participants. Participants lay on the scanner table in the supine position wearing light clothing without any metal objects (jewelry, zippers, belts, snaps, etc.) with no shoes. A posterior-anterior scan image was obtained by whole-body DXA. Arm, leg, and head regions in the scan image were isolated from the trunk region in accordance with the standard manufacturer-recommended methods using the following anatomical landmarks: (a) the horizontal neck line just below the chin, (b) the vertical line bisecting the shoulder joints and (c) the lower pelvic divider lines (two angled lines) bisecting both femoral necks [16]. Body weight and height were measured in light clothing with no shoes. FM and LM indices were calculated as FM and LM divided by height squared (kg/m^2^). BMI was calculated as body weight divided by height squared (kg/m^2^). Overweight and underweight participants were identified using BMI cut-offs of 25 kg/m^2^ and 18.5 kg/m^2^, respectively. Percent body fat (%) was calculated as FM divided by body weight. TAR was calculated as trunk fat divided by appendicular fat (sum of arm fat and leg fat), and TLR as trunk fat divided by leg fat. FM-to-LM ratio was calculated as FM divided by LM.

### Identification of hypertension, dyslipidemia and diabetes

Hypertension was identified as systolic blood pressure ≥140 mmHg and/or diastolic blood pressure ≥90 mmHg [21] and/or the use of antihypertensive drugs. Dyslipidemia was identified as the use of antidyslipidemic drugs and/or triglycerides (TG) >150 mg/dL and/or low-density lipoprotein (LDL) cholesterol >140 mg/dl and/or high-density lipoprotein (HDL) cholesterol <40 mg/dl [22] in overnight fasting samples, or LDL cholesterol >140 mg/dl and/or HDL cholesterol <40 mg/dl in non-overnight fasting samples. Diabetes was identified as hemoglobin A1c (National Glycohemoglobin Standardization Program) ≥6.5% [23] and/or the use of antidiabetic drugs.

### Measurements of blood pressure and blood samples

Blood pressure measurement was performed using an automatic oscillometric sphygmomanometer (BP-203i, OMRON COLIN, Tokyo, Japan) with an appropriate cuff size. Participants were seated with legs uncrossed at an appropriate ambient temperature for 5 minutes. Two measurements were performed with the right arm supported at the level of the heart. The mean value of two records was used for analysis.

The following methods were used to determine blood marker levels: TG, glycerol phosphate oxidase with glycerol blank, Pureauto S TG-N, Sekisui Medical Co., Ltd., Tokyo, Japan; HDL cholesterol, Sekisui HDL direct method, Cholestest N HDL, Sekisui Medical Co., Ltd., Tokyo, Japan; LDL cholesterol, Sekisui LDL direct method, Cholestest LDL, Sekisui Medical Co., Ltd., Tokyo, Japan; total cholesterol, ultraviolet method with cholesterol dehydrogenase, T-CHO reagents, KL and “Kokusai”, Sysmex Corp., Kobe, Japan; fasting plasma glucose, ultraviolet method with hexokinase, CicaLiquid GLU, Kanto Chemical Co., Inc., Tokyo, Japan; hemoglobin A1c, National Glycohemoglobin Standardization Program, latex aggregation immunoassay, RAPIDIA Auto HbA1c, Fujirebio Inc., Tokyo, Japan; and fasting serum insulin, chemiluminescent enzyme immunoassay, Lumipulse Presto Insulin, Fujirebio Inc., Tokyo, Japan. Homeostasis model assessment-insulin resistance (HOMA-IR) was calculated using fasting plasma glucose and fasting serum insulin [24].

### Lifestyle and medications

Participants were interviewed by trained health care nurses using a self-administered lifestyle questionnaire including smoking (current or non-smoker) and physical activity items. Physical activity was assessed using the Japanese version of the International Physical Activity Questionnaire (IPAQ), which has been validated for adults aged 65 years and older [25]. Metabolic equivalent of task (MET)-minutes/week was estimated in accordance with official IPAQ guidelines [26]. Participants also brought current medications, and the nurses recorded the name and dose of each medication. The Food Frequency Questionnaire for the Prevention and Management of Osteoporosis (FFQPOP) [27] was used to estimate dietary nutrient intake during the past one month. Responses to the FFQPOP were verified by dietitians with participants. The Standard Table of Food Composition in Japan [28] was used to estimate nutrient and energy intake.

### Statistical analysis

We confirmed log-normal frequency distributions of biochemical blood tests prior to statistical analyses. Accordingly, values were logarithmically converted, analyzed statistically and expressed as geometric means. All statistical analyses were performed with IBM SPSS statistics Version 26. P < 0.05 was considered statistically significant.

Differences between participants with and without cardiometabolic diseases were assessed using the unpaired t-test, Mann–Whitney U test or Fisher’s exact test. Participants were divided according to DXA-measured body composition indices into five equally sized groups (quintiles [Q] 1 to 5). Odds ratios (ORs) for cardiometabolic diseases in Q2–Q5 versus Q1, and in Q1, Q3, Q4 and Q5 versus Q2 were calculated using multivariate logistic regression analysis after adjusting for potential confounding variables including age, MET-minutes/week, current smoker, alcohol intake and energy intake. Variables which may affect relationships between DXA-based indices (predictors) and cardiometabolic disease (outcome), such as age, were selected as potential confounding factors. The ability of DXA-based indices to discriminate between the presence and absence cardiometabolic diseases was evaluated using receiver operating characteristic (ROC) curve analysis [29]. The area under the curve (AUC) was used to quantify the discriminative ability of DXA-based indices [29]. An AUC significantly (P < 0.05) larger than 0.5 was considered satisfactory performance. Differences in the discriminative ability of DXA-based indices were considered statistically significant if the 95% confidence intervals (CIs) of the AUCs did not overlap. To assess associations among DXA-based indices, Pearson’s correlation coefficients were calculated.

## Results

Table 1 shows differences between participants with and without cardiometabolic diseases (hypertension, dyslipidemia and diabetes). The FM index, percent body fat and FM-to-LM ratio were higher in participants with hypertension or dyslipidemia than in those without, whereas no significant differences were observed between participants with and without diabetes. On the other hand, TAR and TLR were significantly higher in participants with hypertension, dyslipidemia or diabetes than in those without. No significant difference was observed in the LM index between participants with and without any of the cardiometabolic diseases examined.

**Table 1:** Differences between participants with and without cardiometabolic diseases.

	**Hypertension**			**Dyslipidemia**			**Diabetes**		
		
	**With, N = 411**	**Without, N = 184**	**P**	**With, N = 322**	**Without, N = 273**	**P**	**With, N = 125**	**Without, N = 470**	**P**
Age (years)	73.9 ± 5.4	72.2 ± 5.0	<0.01	72.9 ± 5.3	73.8 ± 5.4	0.04	73.4 ± 4.6	73.3 ± 5.6	ns
Height (cm)	164.5 ± 5.9	165.5 ± 5.6	ns	165.5 ± 5.8	164.0 ± 5.7	<0.01	164.8 ± 5.3	164.8 ± 5.9	ns
Weight (kg)	63.7 ± 8.7	62.4 ± 7.9	ns	64.6 ± 8.1	61.8 ± 8.6	<0.01	64.0 ± 8.9	63.1 ± 8.4	ns
Body mass index (kg/m^2^)	23.5 ± 2.8	22.8 ± 2.6	<0.01	23.6 ± 2.5	23.0 ± 2.9	0.01	23.5 ± 2.9	23.2 ± 2.7	ns
Whole-body fat (kg)	13.5 ± 4.7	11.9 ± 4.6	<0.01	13.8 ± 4.4	12.1 ± 4.8	<0.01	13.0 ± 5.0	13.0 ± 4.6	ns
Trunk fat (kg)	7.2 ± 2.9	6.1 ± 2.9	<0.01	7.4 ± 2.8	6.2 ± 3.0	<0.01	7.0 ± 3.1	6.8 ± 2.9	ns
Appendicular fat (kg)	5.6 ± 2.0	5.0 ± 1.8	<0.01	5.6 ± 1.9	5.1 ± 2.0	<0.01	5.2 ± 2.1	5.5 ± 1.9	ns
Leg fat (kg)	4.2 ± 1.5	3.7 ± 1.4	<0.01	4.2 ± 1.4	3.8 ± 1.5	<0.01	3.8 ± 1.5	4.1 ± 1.4	ns
FM index (kg/m^2^)	5.0 ± 1.7	4.3 ± 1.7	<0.01	5.0 ± 1.6	4.5 ± 1.8	<0.01	4.8 ± 1.8	4.8 ± 1.7	ns
Percent body fat (%)	21.9 ± 5.6	19.5 ± 5.9	<0.01	22.1 ± 5.3	20.1 ± 6.3	<0.01	20.9 ± 6.1	21.2 ± 5.7	ns
TAR	1.3 ± 0.4	1.2 ± 0.4	<0.01	1.3 ± 0.3	1.2 ± 0.4	<0.01	1.4 ± 0.4	1.2 ± 0.3	<0.01
TLR	1.8 ± 0.6	1.6 ± 0.5	<0.01	1.8 ± 0.5	1.6 ± 0.6	<0.01	1.9 ± 0.7	1.7 ± 0.5	<0.01
LM index (kg/m^2^)	16.6 ± 1.4	16.6 ± 1.3	ns	16.6 ± 1.4	16.6 ± 1.5	ns	16.8 ± 1.6	16.6 ± 1.4	ns
FM-to-LM ratio	0.30 ± 0.10	0.26 ± 0.10	<0.01	0.30 ± 0.09	0.27 ± 0.11	<0.01	0.29 ± 0.10	0.29 ± 0.10	ns
Alcohol intake (kcal/day)	97 ± 145	77 ± 118	ns	78 ± 117	106 ± 157	0.02	68 ± 92	97 ± 146	<0.01
Energy intake (kcal/day)	1689 ± 319	1730 ± 291	ns	1711 ± 288	1691 ± 335	ns	1688 ± 339	1705 ± 303	ns
NaCl intake (g/day)	10.0 ± 1.8	10.2 ± 2.0	ns	10.0 ± 1.8	10.1 ± 1.9	ns	10.0 ± 1.7	10.0 ± 1.9	ns
Systolic blood pressure (mmHg)	145.2 ± 16.8	125.6 ± 10.8	<0.01	140.0 ± 18.0	138.2 ± 17.4	ns	136.8 ± 17.5	139.7 ± 17.7	ns
Diastolic blood pressure (mmHg)	80.9 ± 10.5	73.9 ± 7.9	<0.01	79.8 ± 10.5	77.5 ± 9.9	<0.01	75.3 ± 10.3	79.7 ± 10.1	<0.01
LDL cholesterol (mg/dl)	114.4 ^×^/_÷_ 1.3	115.1 ^×^/_÷_ 1.3	ns	123.9 ^×^/_÷_ 1.3	104.6 ^×^/_÷_ 1.3	<0.01	104.8 ^×^/_÷_ 1.3	117.4 ^×^/_÷_ 1.3	<0.01
HDL cholesterol (mg/dl)	60.9 ^×^/_÷_ 1.3	62.4 ^×^/_÷_ 1.3	ns	59.0 ^×^/_÷_ 1.3	64.3 ^×^/_÷_ 1.3	<0.01	60.0 ^×^/_÷_ 1.3	61.8 ^×^/_÷_ 1.3	ns
Total cholesterol (mg/dl)	201.5 ^×^/_÷_ 1.2	203.2 ^×^/_÷_ 1.2	ns	210.4 ^×^/_÷_ 1.2	192.5 ^×^/_÷_ 1.1	<0.01	191.1 ^×^/_÷_ 1.2	205.0 ^×^/_÷_ 1.2	<0.01
Triglycerides (mg/dl)	97.8 ^×^/_÷_ 1.6	94.8 ^×^/_÷_ 1.6	ns	109.9 ^×^/_÷_ 1.6	83.4 ^×^/_÷_ 1.6	<0.01	110.0 ^×^/_÷_ 1.6	93.6 ^×^/_÷_ 1.6	<0.01
Hemoglobin A1c (%)	5.9 ^×^/_÷_ 1.1	5.9 ^×^/_÷_ 1.1	ns	5.9 ^×^/_÷_ 1.1	5.9 ^×^/_÷_ 1.1	ns	6.9 ^×^/_÷_ 1.1	5.6 ^×^/_÷_ 1.1	<0.01
Fasting serum insulin (mU/l)	4.4 ^×^/_÷_ 2.0	3.9 ^×^/_÷_ 2.0	ns	4.7 ^×^/_÷_ 1.8	3.8 ^×^/_÷_ 2.1	<0.01	6.3 ^×^/_÷_ 2.5	3.8 ^×^/_÷_ 1.8	<0.01
Fasting plasma glucose (mg/dl)	103.6 ^×^/_÷_ 1.3	100.5 ^×^/_÷_ 1.2	ns	102.5 ^×^/_÷_ 1.2	102.9 ^×^/_÷_ 1.3	ns	135.3 ^×^/_÷_ 1.4	95.4 ^×^/_÷_ 1.1	<0.01
HOMA-IR	1.1 ^×^/_÷_ 2.3	1.0 ^×^/_÷_ 2.3	<0.01	1.2 ^×^/_÷_ 2.0	1.0 ^×^/_÷_ 2.5	<0.01	2.1 ^×^/_÷_ 3.0	0.9 ^×^/_÷_ 1.9	<0.01
MET-minutes/week, median (25, 75%ile)	2310 (990, 4326)	2099 (1098, 4529)	ns	2079 (990, 4203)	2568 (1087, 5001)	ns	2079 (1134, 4487)	2264 (1010, 4374)	ns
Overweight, N (%)	113 (27)	37 (20)	ns	78 (24)	72 (20)	ns	32 (25)	118 (25)	ns
Underweight, N (%)	13 (3)	11 (6)	ns	7 (2)	17 (6)	0.02	4 (3)	20 (4)	ns
Current smoker, N (%)	39 (9)	17 (9)	ns	31 (10)	25 (9)	ns	13 (10)	43 (9)	ns

Data are expressed as mean ± SD, median (25, 75%ile), geometric mean ^×^/_÷_ SD or N (%).

Biochemical marker values were logarithmically converted and then statistically analyzed.

P-values were calculated using the unpaired t-test, Mann–Whitney U test or Fisher’s exact test. P < 0.05 was considered statistically significant.

MET-minutes/week was calculated using the International Physical Activity Questionnaire.

N, number; ns, not significant; FM, fat mass; TAR, trunk-to-appendicular fat ratio; TLR, trunk-to-leg fat ratio; LM, lean mass; LDL, low-density lipoprotein; HDL, high-density lipoprotein; HOMA-IR, homeostasis model assessment-insulin resistance; MET, metabolic equivalent of task; SD, standard deviation.

Table 2 shows ORs for cardiometabolic diseases in Q2–Q5 versus Q1, and Table 3 shows ORs for cardiometabolic diseases in Q1, Q3, Q4 and Q5 versus Q2. Except for the LM index, all DXA-based indices (i.e., FM index, percent body fat, TAR, TLR and FM-to-LM ratio) were significantly associated with hypertension and dyslipidemia. The FM index and percent body fat showed no positive association with diabetes, whereas TAR and TLR showed significant positive associations with diabetes. There were no significant associations between the LM index and cardiometabolic risk factors including hypertension, dyslipidemia and diabetes.

**Table 2:** aORs for cardiometabolic diseases in Q2–Q5 versus Q1.

	**Hypertension**		**Dyslipidemia**		**Diabetes**	
		
	**aOR**	**P**	**aOR**	**P**	**aOR**	**P**
Body mass index Q1, reference	1.00		1.00		1.00	
Body mass index Q2	1.08	ns	2.80	<0.01	1.24	ns
Body mass index Q3	1.61	ns	3.11	<0.01	1.28	ns
Body mass index Q4	2.18	<0.01	2.31	<0.01	0.86	ns
Body mass index Q5	1.95	0.02	1.92	0.02	1.19	ns

FM index Q1, reference	1.00		1.00		1.00	
FM index Q2	1.92	0.02	2.04	<0.01	0.98	ns
FM index Q3	2.25	<0.01	2.14	<0.01	0.79	ns
FM index Q4	3.28	<0.01	2.03	<0.01	0.77	ns
FM index Q5	3.32	<0.01	2.57	<0.01	0.90	ns

Percent body fat Q1, reference	1.00		1.00		1.00	
Percent body fat Q2	1.61	ns	1.74	0.04	0.82	ns
Percent body fat Q3	2.29	<0.01	1.93	0.01	1.07	ns
Percent body fat Q4	3.16	<0.01	2.28	<0.01	0.48	0.04
Percent body fat Q5	3.10	<0.01	2.35	<0.01	1.13	ns

TAR Q1, reference	1.00		1.00		1.00	
TAR Q2	1.43	ns	2.00	0.01	1.31	ns
TAR Q3	1.30	ns	1.48	0.14	1.34	ns
TAR Q4	1.85	0.04	2.92	<0.01	1.86	ns
TAR Q5	1.74	ns	2.16	<0.01	2.34	0.01

TLR Q1, reference	1.00		1.00		1.00	
TLR Q2	1.29	ns	1.37	ns	0.94	ns
TLR Q3	1.18	ns	1.60	ns	1.55	ns
TLR Q4	1.93	0.03	1.97	0.01	1.35	ns
TLR Q5	1.68	ns	2.10	<0.01	2.21	0.02

LM index Q1, reference	1.00		1.00		1.00	
LM index Q2	0.88	ns	1.07	ns	1.13	ns
LM index Q3	1.27	ns	1.38	ns	1.02	ns
LM index Q4	0.91	ns	1.30	ns	1.12	ns
LM index Q5	1.03	ns	1.02	ns	1.46	ns

FM-to-LM ratio Q1, reference	1.00		1.00		1.00	
FM-to-LM ratio Q2	1.60	ns	1.70	0.05	0.78	ns
FM-to-LM ratio Q3	2.37	<0.01	1.88	0.02	1.06	ns
FM-to-LM ratio Q4	2.85	<0.01	2.04	<0.01	0.52	ns
FM-to-LM ratio Q5	3.28	<0.01	2.35	<0.01	1.13	ns

Multivariate logistic regression analysis was used to calculate aORs, adjusted for age, MET-minutes/week, current smoker, alcohol intake and energy intake. P < 0.05 was considered statistically significant.

aOR, adjusted odds ratio; Q, quintile; ns, not significant; FM, fat mass; TAR, trunk-to-appendicular fat ratio; TLR, trunk-to-leg fat ratio; LM, lean mass.

**Table 3:** aORs for cardiometabolic diseases in Q1, Q3, Q4 and Q5 versus Q2.

	**Hypertension**		**Dyslipidemia**		**Diabetes**	
		
	**aOR**	**P**	**aOR**	**P**	**aOR**	**P**
Body mass index Q1	0.92	ns	0.36	<0.01	0.81	ns
Body mass index Q2, reference	1.00		1.00		1.00	
Body mass index Q3	1.48	ns	1.11	ns	1.03	ns
Body mass index Q4	2.01	0.01	0.83	ns	0.70	ns
Body mass index Q5	1.81	0.04	0.69	ns	0.96	ns

FM index Q1	0.52	0.02	0.49	<0.01	1.02	ns
FM index Q2, reference	1.00		1.00		1.00	
FM index Q3	1.17	ns	1.05	ns	0.80	ns
FM index Q4	1.71	ns	0.99	ns	0.78	ns
FM index Q5	1.73	ns	1.26	ns	0.91	ns

Percent body fat Q1	0.62	ns	0.57	0.04	1.23	ns
Percent body fat Q2, reference						
Percent body fat Q3	1.42	ns	1.11	ns	1.31	ns
Percent body fat Q4	1.97	0.02	1.31	ns	0.59	ns
Percent body fat Q5	1.93	0.03	1.35	ns	1.39	ns

TAR Q1	0.70	ns	0.50	0.01	0.76	ns
TAR Q2, reference	1.00		1.00		1.00	
TAR Q3	0.91	ns	0.74	ns	1.02	ns
TAR Q4	1.30	ns	1.46	ns	1.42	ns
TAR Q5	1.22	ns	1.08	ns	1.78	ns

TLR Q1	0.77	ns	0.73	ns	1.06	ns
TLR Q2, reference	1.00		1.00		1.00	
TLR Q3	0.91	ns	1.17	ns	1.65	ns
TLR Q4	1.50	ns	1.44	ns	1.43	ns
TLR Q5	1.30	ns	1.53	ns	2.35	<0.01

LM index Q1	1.14	ns	0.94	ns	0.89	ns
LM index Q2, reference	1.00		1.00		1.00	
LM index Q3	1.44	ns	1.29	ns	0.91	ns
LM index Q4	1.03	ns	1.21	ns	0.99	ns
LM index Q5	1.18	ns	0.96	ns	1.29	ns

FM-to-LM ratio Q1	0.62	ns	0.59	0.05	1.28	ns
FM-to-LM ratio Q2, reference	1.00		1.00		1.00	
FM-to-LM ratio Q3	1.48	ns	1.11	ns	1.35	ns
FM-to-LM ratio Q4	1.78	ns	1.20	ns	0.67	ns
FM-to-LM ratio Q5	2.05	0.02	1.38	ns	1.45	ns

Multivariate logistic regression analysis was used to calculate aORs, adjusted for age, MET-minutes/week, current smoker, alcohol intake, and energy intake. P < 0.05 was considered statistically significant.

aOR, adjusted odds ratio; Q, quintile; ns, not significant; FM, fat mass; TAR, trunk-to-appendicular fat ratio; TLR, trunk-to-leg fat ratio; LM, lean mass.

Table 4 shows the ability of DXA-based indices to discriminate between the presence and absence of cardiometabolic diseases. AUCs were significantly larger than 0.5 for the FM index, percent body fat, TAR, TLR and FM-to-LM ratio for patients with hypertension and those with dyslipidemia, and for TAR and TLR in patients with diabetes. No significant AUCs were obtained for the LM index. The upper limit of the 95% CI for the LM index in the AUC for hypertension was lower than the lower limit for the FM index and percent body fat. Thus, a statistically significant difference was found between the LM index and the FM index in the ability to discriminate between the presence and absence of hypertension. Similarly, the LM index had a significantly lower ability to discriminate between the presence and absence of hypertension than the FM-to-LM ratio.

**Table 4:** Ability of DXA-based indices to discriminate between the presence and absence of cardiometabolic diseases.

	**Hypertension**				**Dyslipidemia**				**Diabetes**			
		
	**AUC**	**P**	**95%CI**		**AUC**	**P**	**95%CI**		**AUC**	**P**	**95%CI**	
		
			**Lower**	**Upper**			**Lower**	**Upper**			**Lower**	**Upper**
Body mass index	0.58	<0.01	0.53	0.63	0.55	ns	0.50	0.59	0.51	ns	0.45	0.56
FM index	0.62	<0.01	0.57	0.67	0.58	<0.01	0.53	0.63	0.49	ns	0.43	0.54
Percent body fat	0.62	<0.01	0.57	0.67	0.59	<0.01	0.54	0.64	0.49	ns	0.43	0.55
TAR	0.56	0.02	0.51	0.61	0.58	<0.01	0.54	0.63	0.58	<0.01	0.53	0.64
TLR	0.56	0.02	0.51	0.61	0.58	<0.01	0.54	0.63	0.58	<0.01	0.52	0.64
LM index	0.50	ns	0.45	0.55	0.51	ns	0.46	0.56	0.52	ns	0.47	0.58
FM-to-LM ratio	0.62	<0.01	0.57	0.67	0.59	<0.01	0.54	0.64	0.49	ns	0.43	0.55

AUCs were calculated using receiver operating characteristic curve analysis.

P < 0.05 was considered statistically significant.

AUC, area under the curve; CI, confidence intervals; ns, not significant; Lower, lower limt; Upper, upper limit; FM, fat mass; TAR, trunk-to-appendicular fat ratio; TLR, trunk-to-leg fat ratio; LM, lean mass.

Table 5 shows Pearson’s correlation coefficients among DXA-measured body composition indices. The FM index and percent body fat showed very strong correlations with FM-to-LM ratio. The LM index showed no significant correlation with FM-to-LM ratio or percent body fat, and weak correlations with the FM index, TAR and TLR. TAR and TLR showed weak correlations with the FM index and percent body fat.

**Table 5:** Associations among DXA-measured body composition indices.

	**Percent body fat**		**TAR**		**TLR**		**LM index**		**FM-to-LM ratio**	
				
	**r**	**P**	**r**	**P**	**r**	**P**	**r**	**P**	**r**	**P**
FM index	0.97	<0.01	0.39	<0.01	0.36	<0.01	0.23	<0.01	0.97	<0.01
Percent body fat			0.39	<0.01	0.35	<0.01	0.01	ns	0.99	<0.01
TAR					0.98	<0.01	0.22	<0.01	0.36	<0.01
TLR							0.23	<0.01	0.32	<0.01
LM index									0.00	ns

r: Pearson’s correlation coefficient was assessed. P < 0.05 was considered statistically significant.

TAR, trunk-to-appendicular fat ratio; TLR, trunk-to-leg fat ratio; LM, lean mass; ns, not significant.

## Discussion

In the present community-based, single-center, cross-sectional study targeting Japanese elderly men, DXA-based indices such as the FM index, percent body fat, TAR, TLR and FM-to-LM ratio were significantly associated with hypertension and dyslipidemia. In addition, TAR and TLR showed significant positive associations with diabetes, whereas the FM index, percent body fat and FM-to-LM ratio showed no positive associations with diabetes. BMI and FM index are commonly used weight-based adiposity measures that do not provide information on body fat distribution. In contrast, waist-to-hip circumference ratio, TAR and TLR are fat distribution-based measures [18]. The present findings indicate that fat distribution-based measures (i.e., TAR and TLR) may provide additional information that weight-based measures (i.e., FM index and percent body fat) do not offer, especially for the management of diabetes. However, there are only a limited number of facilities that can perform DXA body composition measurements, and the costs associated with measurements are high in clinical settings. On the other hand, skeletal muscle included in LM has many metabolic functions and is an emerging factor for cardiovascular diseases [11]. Previous reports have suggested that the loss of skeletal muscle mass may contribute to the development of metabolic diseases [14]. However, both logistic and ROC curve analyses in the present study population revealed no associations between the LM index and cardiometabolic diseases, suggesting that the association between cardiometabolic function and LM, which includes skeletal muscle, may not be as pronounced or stronger than associations between cardiometabolic function and total and regional FM-related parameters. Further detailed studies are needed to clarify how skeletal muscle contributes to cardiometabolic disease.

The FM index and percent body fat did not differ significantly between participants with and without diabetes, while significant differences were observed for TAR and TLR. TAR is calculated as trunk fat divided by appendicular fat (sum of arm fat and leg fat), and TLR as trunk fat divided by leg fat. Trunk fat includes both visceral fat and subcutaneous fat, whereas peripheral (i.e., arm or leg) fat includes only subcutaneous fat. Thus, trunk-to-extremity (peripheral) fat ratios such as TAR and TLR may reveal the relative impact of visceral to subcutaneous fat with regard to cardiometabolic risks [30]. Visceral adipose tissue reportedly is an independent risk marker of cardiovascular and metabolic morbidity and mortality [3], whereas accumulation of fat in the lower body (gluteofemoral obesity) has been suggested to be associated with a low risk of cardiovascular disease and type 2 diabetes mellitus when adjusted for overall fat mass [6].

Importantly, some individuals who are not obese on the basis of height and weight can be hyperinsulinemic, insulin-resistant and predisposed to type 2 diabetes, hypertriglyceridemia and premature coronary heart disease, like people with overt obesity [31]. Such metabolically obese, normal-weight (MONW) individuals are very common in the general population [31] and are often characterized as having excess visceral adipose tissue and ectopic fat deposition [32]. Japanese men, in particular, have larger areas of visceral adipose tissue than Caucasian men, and are less obese but more susceptible to metabolic disorders [4, 5]. Trunk-to-peripheral fat ratio measured by DXA may help characterize the profiles of MONW individuals [30]. In the present study, participants with diabetes did not show significantly larger FM index or percent body fat values compared with non-diabetic participants. The present findings suggested that some participants with diabetes were MONW individuals, and that TAR and TLR, i.e., trunk-to-peripheral fat ratios, may be useful as indices for characterizing this particular population, given the high prevalence of the MONW phenotype in Asian populations.

The present study has potential limitations. First, participants were not randomly selected, and thus may not be fully representative of the Japanese elderly population. Moreover, only elderly males were included in the study. Therefore, caution should be exercised in generalizing the results. Second, due to the cross-sectional design, we were unable to investigate the longitudinal relationship between DXA-based indices and cardiometabolic diseases. Third, LM is calculated under the assumption that the water content in LM is generally about 73% in DXA examinations. However, the participants of this study were men aged 65 years or older, and thus the water content in LM may be less than that in the general population, which could lead to the underestimation of LM [33].

## Conclusion

In the present study targeting Japanese elderly men, DXA-based indices such as the FM index, percent body fat, TAR, TLR and FM-to-LM ratio were significantly associated with hypertension and dyslipidemia. TAR and TLR, but not the FM index, percent body fat and FM-to-LM ratio, were significantly positively associated with diabetes. The LM index had a significantly lower ability to discriminate between the presence and absence of hypertension than the FM index, percent body fat and the FM-to-LM ratio. No associations were observed between the LM index and hypertension, dyslipidemia and diabetes. These results suggest that the effects of LM on cardiometabolic disease may not be as pronounced or as strong as those of FM. Further detailed studies are needed to clarify the effects of skeletal muscle on cardiometabolic diseases.

## Abbreviations

aOR: adjusted odds ratio
AUC: area under the curve
BMI: body mass index
DXA: dual-energy X-ray absorptiometry
FFQPOP: Food Frequency Questionnaire for the Prevention and Management of Osteoporosis
FM: fat mass
FORMEN: Fujiwara-kyo Osteoporosis Risk in Men
HDL: high-density lipoprotein
HOMA-IR: Homeostasis model assessment-insulin resistance
IPAQ: International Physical Activity Questionnaire
LDL: low-density lipoprotein
LM: lean mass
MET: metabolic equivalent of task
MONW: metabolically obese, normal-weight
N: number
ns: not significant
ORs: odds ratios
Q: quintile
ROC: receiver operating characteristic
SD: standard deviation
TAR: trunk-to-appendicular fat ratio
TG: triglycerides
TLR: trunk-to-leg fat ratio

## Declarations

### Availability of data and materials

The data that support the findings of the present study are available on request from the corresponding author KK.
